# Efficacy and safety of intravenous tenecteplase thrombolysis in diffusion-weighted imaging-negative posterior circulation ischemic stroke

**DOI:** 10.3389/fneur.2025.1633214

**Published:** 2025-10-21

**Authors:** Ying Zhang, Shengqi Fu, Shengjie Hu, Lili Zhu, Jiarong Li, Baoyang Shi, Liang Song, Dongdong Yang

**Affiliations:** Department of Neurology, The Fifth Clinical Medical College of Henan University of Chinese Medicine (Zhengzhou People’s Hospital), Zhengzhou, China

**Keywords:** posterior circulation ischemic stroke, diffusion-weighted imaging, negative, early deterioration of neurological function, thrombolytic therapy, propensity score matching

## Abstract

**Introduction:**

Clear evidence supporting thrombolytic therapy in diffusion-weighted imaging (DWI)-negative posterior circulation ischemic stroke (PCIS) is lacking. We aimed to investigate the efficacy and safety of intravenous thrombolysis using tenecteplase (TNK) for the treatment of DWI-negative PCIS.

**Method:**

A retrospective analysis was conducted on 310 patients with DWI-negative PCIS (TNK group, 100 patients; control group, 210 patients) with propensity score matching (PSM, 63 pairs). Efficacy was assessed using the 90-day modified Rankin Scale (mRS) score and early neurological deterioration (END); safety was evaluated by mortality and symptomatic intracerebral hemorrhage (sICH).

**Results:**

The PSM-matched cohort comprised 126 patients (67 men), with a mean age of 68.9 ± 7.9 years. After PSM matching, the 24 h National Institutes of Health Stroke Scale (NIHSS) scores of the two patient groups [3.0 (3.0, 5.0) vs. 4.0 (3.0, 6.0) points] and the NIHSS scores at discharge [2.0 (1.0, 3.0) vs. 3.0 (2.0, 4.0) points] (*p* < 0.05) were compared. In the PSM-matched TNK group, the 90-day 0–1 mRS score (85.7% vs. 58.7%, *p* = 0.028) and END rate (1.6% vs. 19.0%, *p* = 0.011) were significantly better than those of the control group with no increased mortality or sICH. However, the control group had a 90-day mortality rate of 3.2% (2/63; both patients died of stroke-induced pulmonary infections).

**Conclusion:**

In patients with DWI-negative PCIS, TNK increased the proportion of patients achieving a mRS score of 0–1, reduced the incidence of END, improved long-term prognosis, and demonstrated a favorable safety profile; in contrast, the control group exhibited a higher incidence of END and poorer overall prognosis. Notably, this study has limitations, including its single-center retrospective design and small sample size after PSM, which may restrict the generalizability of the present findings.

## 1 Introduction

Posterior circulation ischemic stroke (PCIS) is the narrowing, occlusion, or *in situ* thrombosis of the posterior circulation arteries, causing insufficient blood flow to the brain tissue and leading to ischemic and hypoxic damage, accounting for 20%–25% of all ischemic strokes (1). Diffusion-weighted imaging (DWI), a magnetic resonance imaging (MRI) sequence, of the head is crucial for detecting early ischemic lesions and evaluating acute ischemic stroke (AIS). However, approximately 50% of patients with acute PCIS do not show obvious ischemic lesions on DWI within 24 h of onset. Additionally, the posterior circulatory system supplies a large area of the brain tissue. Therefore, during an infarction, the clinical symptoms are diverse and have low specificity. The commonly used prehospital stroke scoring scale has limited utility in fully evaluating PCIS, leading to a challenging clinical diagnosis, delayed treatment, and high disability and mortality rates (2, 3). Currently, PCIS treatment in clinical practice is controversial. Dual antiplatelet therapy with clopidogrel and aspirin has been shown to be ineffective (4). Intravenous thrombolysis within a particular time window is a preferred treatment method for patients with AIS. However, considering the challenges in early clinical diagnosis of PCIS combined with its subtle neurological deficits, intravenous thrombolysis therapy, which can cause early neurological deterioration (END), is rarely performed (5). In the Trace-2 study, two groups of patients with AIS not eligible for endovascular therapy were treated within 4.5 h with tenecteplase (TNK) and alteplase. The results showed that the efficacy of intravenous thrombolysis with TNK was similar to that of alteplase, with no significant difference in safety outcomes between the two groups (6). In this study, we performed TNK intravenous thrombolysis aiming to investigate the efficacy and safety of early intravenous thrombolysis in patients with DWI-negative PCIS and provide new ideas and methods for the treatment of such patients in clinical practice. Head Impulse Nystagmus Test of Skew (HINTS) has been shown to increase PCIS diagnostic rate (7). Therefore, we also included patients with clinical manifestations of acute PCIS who tested positive for HINTS.

## 2 Materials and methods

### 2.1 Research object

We retrospectively collected data from 310 patients with PCIS admitted to the Stroke Center of Zhengzhou People’s Hospital between August 2018 and May 2024. The inclusion criteria comprised (1) meeting the diagnostic criteria for AIS in the Chinese Guidelines for the Diagnosis and Treatment of Acute Ischemic Stroke 2023 (2); (2) the time between symptom onset and hospital arrival was ≤ 4.5 h; (3) individuals aged ≥ 18 years or older; (4) neurological deficit symptoms caused by ischemic stroke, with a National Institutes of Health Stroke Scale (NIHSS) score of ≤5 upon admission; (5) positive HINTS or HINTS PLUS examination; (6) Cerebral infarction with complete head CT (Computed Tomography) and MRI (Magnetic Resonance Imaging) examinations, and negative findings on MRI DWI (Diffusion-Weighted Imaging) sequences; and (7) participants who had been informed of the study’s purpose and had signed informed consent forms. The exclusion criteria were as follow: (1) acute anterior circulation stroke, transient ischemic attack, and hemorrhagic and watershed stroke; (2) history of cardiac, pulmonary, hepatic, renal, or other major organ dysfunction, as well as psychiatric disorders; (3) inability to complete head CT + MRI examination; (4) positive DWI sequence; (5) pregnant or lactating women; (6) history of previous intracranial hemorrhage; (7) intracranial tumors, arteriovenous malformations, and aneurysms; and (8) coagulation dysfunction, acute bleeding tendency, active internal bleeding, and platelet count < 100 × 10^9^/L. This study was approved by the Ethics Committee of the Zhengzhou People’s Hospital (approval number: 2018006015).

### 2.2 Data

We documented demographic characteristics and cardiovascular risk profiles, including age, sex, smoking status, hypertension, diabetes mellitus, dyslipidemia, history of cerebrovascular events, coronary artery disease, and atrial fibrillation; baseline systolic and diastolic blood pressure; MRI re-examination at 48–72 h to record the location of the patient’s infarction; and laboratory tests, including triglycerides, total cholesterol, high-density lipoprotein cholesterol (HDL-C), low-density lipoprotein cholesterol (LDL-C), homocysteine (HCY), fasting plasma glucose (FPG), and the NIHSS score at admission.

### 2.3 Grouping

We enrolled 310 patients with PCIS who had negative MRI-DWI sequences and classified them into a thrombolysis group of 100 patients and a control group of 210 patients based on whether they received intravenous thrombolysis treatment. Patients in the thrombolysis group were treated for intravenous thrombolysis using the standard dose of TNK 0.25 mg/kg (produced by Guangzhou Mingkang Bioengineering Co., Ltd., National Medical Products Standard S20150001, specification: 1.0 × 10 E7IU/16 mg per tube), with a maximum dose not exceeding 25 mg. The medication was administered as a single intravenous injection within 5–10 s. The patients were then administered clopidogrel 75 mg/day + aspirin 100 mg/day combined with antiplatelet aggregation treatment after 24 h of head CT or MRI examination to exclude intracranial hemorrhage for 21 days. Subsequently, clopidogrel 75 mg/day or aspirin 100 mg/day were administered as a single antiplatelet aggregation treatment. Upon admission, patients in the control group were treated with clopidogrel 300 mg + aspirin 100 mg and subsequently received antiplatelet aggregation therapy following the protocol used for the thrombolysis group.

### 2.4 Imaging data

All participants underwent brain MRI scans using a German-manufactured Siemens MAGNETOM Skyra 3.0 T system equipped with a 12-channel head coil. The imaging protocols included axial T1-weighted (T1WI), T2-weighted (T2WI), T2-fluid-attenuated inversion recovery (FLAIR), and DWI. Scanning sequence and parameters were as follows: T1WI repetition time (TR) 2,400 ms, echo time (TE) 24.0 ms; T2WI TR 3,300 ms, TE 96.0 ms; T2-Flair TR 7,000 ms, TE 94.0 ms; and DWI TR 4,300 ms, TE 109 ms, b values of 0 and 1,000 s/mm^2^. Image quality was evaluated by two neurologists with >5 years of experience in diagnostic imaging, without any communication or access to clinical data. When their opinions differed, they reached a consensus through discussion.

### 2.5 Definition and evaluation of END

END is defined as increase of ≥1 point in the motor score or increase of ≥2 points in the total NIHSS score measured within 7 days compared to the baseline NIHSS score (8). All patients received consecutive NIHSS assessments at four time points, with the first assessment conducted at admission (as baseline). Subsequent evaluations will be conducted at the following times: (1) 24 h (±6 h), (2) 72 h (±6 h), and (3) day 7 (±6 h). All evaluations were conducted by our attending physicians, who had no knowledge of the patient’s treatment allocation and baseline clinical data. For patients who were discharged before the 7th day or died during the follow-up period. This standardized timing protocol eliminates the variability of patient evaluation intervals and directly reduces the risk of measurement bias related to time. In addition, the blindness of evaluators toward treatment status further reduces subjective evaluation bias, ensuring the reliability of END recognition.

### 2.6 Outcome observation indicators

Regarding the efficacy indicators, the NIHSS scores of the patient groups were evaluated at 24 h, 7 days post-thrombolytic therapy, and discharge. Their neurological function recovery status was observed. The modified Rankin Scale (mRS) score was evaluated through telephone or follow-up interviews after 90 days of illness. An mRS score ≤1 reflects good functional prognosis, mRS ≤ 2 functional improvement, and mRS ≥ 4 poor prognosis. Regarding the safety and efficacy indicators, the incidence of intracranial hemorrhage and 90-day mortality rate in both groups of patients were determined through a combination of imaging examinations (head CT/MRI) and electronic medical record reviews; the definition of symptomatic intracranial hemorrhage (sICH) was consistent with the European Stroke Group Standards woven into the 2023 updated evidence-based recommendations: within 24 h after thrombolytic therapy, head CT or MRI shows new intracranial hemorrhage, accompanied by an increase of ≥4 points in the NIHSS score compared to that at baseline (9). In this study, all patients completed head imaging re-examination and NIHSS score retesting at 24 and 48 h after thrombolysis, respectively, to verify the occurrence of sICH and avoid missed incidence or misjudgment.

### 2.7 Statistical analysis

Data were processed and analyzed using statistical software (SPSS 27.0). The patients’ baseline characteristics were matched using propensity score matching (PSM). The matching factors included sex; age; vascular risk factors (elevated blood pressure, diabetes mellitus, coronary heart disease, atrial fibrillation, previous stroke history, hyperlipidemia, and smoking history); baseline NIHSS score; baseline blood pressure; FPG; HCY; and factors such as total cholesterol, triglycerides, HDL-C, and LDL-C, which may affect the outcome. Individuals assigned to the intravenous thrombolysis group were matched after calculating propensity scores based on matching factors using the caliper method, with a caliper value of 0.1. A 1:1 PSM method was used to screen successfully matched data for subsequent analyses. The aim of PSM was to balance the baseline data of the two distinct clinical groups and mitigate potential confounding effects. Rigorous covariate adjustment was used to reduce bias, and normality analysis of quantitative data was conducted using the Shapiro–Wilk test, where normal distribution indicated conformity. An independent sample *t*-test was used for intergroup comparisons, whereas *M* (*Q*1, *Q*3) was used for non-normally distributed data. The Mann–Whitney *U* test (a nonparametric method) was used for intergroup comparison, while categorical variables are expressed as frequencies and percentages. Intergroup comparisons were conducted using Student’s *t*-test. The long-term prognoses of the two treatment methods were compared using the 90-day mRS score. The dependent variable was whether intravenous thrombolysis was conducted using a binary logistic regression model, and the primary and secondary outcome measures were used as independent variables. The bilateral inspection level was *α* = 0.05.

## 3 Results

### 3.1 Comparison of general clinical data between the patient groups

We included 310 patients comprising 180 men and 130 women, with an age range of 41–91 (67.5 ± 8.5) years. The thrombolysis group without PSM and control group exhibited significant differences in various factors such as history of hypertension, smoking status, baseline blood pressure, total cholesterol, HDL-C, fasting blood glucose, homocysteine levels, baseline NIHSS score, 24 h NIHSS score, 7-day NIHSS score, and discharge NIHSS score (all *p* < 0.05). By contrast, there were no significant differences in the other patient baseline characteristics (all *p* > 0.05). Sixty-three pairs of subjects were successfully matched using PSM, and the baseline characteristics of all matched patients were comparable. Following the matching, the differences in 24 h NIHSS scores and NIHSS scores at discharge between the groups were significant (*p* < 0.05; Table 1).

**Table 1:** General clinical data analysis of two groups of patients with DWI-negative PCIS.

Project	Before PSM				After PSM			
	Thrombolytic group (*n* = 100)	Control group (*n* = 210)	*t*/*c*^2^/*Z-*value	*p-*value	Thrombolytic group (*n* = 63)	Control group (*n* = 63)	*t*/*c*^2^/*Z-*value	*p-*value
Male (*n*, %)	52 (52.0)	128 (61.0)	2.23	0.135	33 (52.4)	34 (54.0)	0.03	0.858
Age (years, *x̄* ± *s*)	68 ± 8	67 ± 9	1.34	0.181	69 ± 8	69 ± 8	0.38	0.703
Vascular risk factors (*n*, %)								
Hypertension	54 (54.0)	150 (71.4)	9.15	0.002	36 (57.1)	44 (69.8)	2.19	0.139
Diabetes	28 (28.0)	80 (38.1)	3.04	0.081	23 (36.5)	24 (38.1)	0.03	0.854
Hyperlipidemia	44 (44.0)	110 (52.4)	1.90	0.168	28 (44.4)	29 (46.0)	0.03	0.858
History of coronary heart disease	25 (25.0)	52 (24.8)	<0.01	0.964	19 (30.2)	22 (34.9)	0.33	0.568
History of stroke	15 (15.0)	26 (12.4)	0.41	0.525	7 (11.1)	13 (20.6)	2.14	0.144
History of atrial fibrillation	9 (9.0)	35 (16.7)	3.27	0.071	9 (14.3)	11 (17.5)	0.238	0.626
Smoke	29 (29.0)	88 (41.9)	4.80	0.028	23 (36.5)	21 (33.3)	0.14	0.709
Baseline blood pressure (mmHg)								
Systolic pressure (*x̄* ± *s*)	143 ± 11	146 ± 10	−2.34	0.020	144 ± 13	147 ± 10	−1.09	0.280
Diastolic pressure (*x̄* ± *s*)	93 ± 13	98 ± 9	−3.89	< 0.001	95 ± 12	95 ± 9	−0.17	0.865
Laboratory indicators								
TC [mmoL/L (*x̄* ± *s*)]	5.2 ± 1.0	5.4 ± 1.0	−2.01	0.045	5.2 ± 1.0	5.1 ± 0.9	0.52	0.607
TG [mmoL/L, M (*Q*_1_, *Q*3)]	1.4 (1.1, 2.0)	1.5 (1.2, 2.1)	−1.70	0.089	1.4 (1.1, 2.1)	1.5 (1.2, 1.7)	−0.35	0.727
LDL-C [mmoL/L, M (*Q*_1_, *Q*3)]	1.6 (1.3, 2.1)	1.5 (1.2, 2.0)	−1.84	0.065	1.6 (1.3, 2.0)	1.7 (1.3, 2.3)	−1.09	0.275
HDL-C [mmoL/L, M (*Q*_1_, *Q*3)]	1.3 (1.1, 1.6)	1.2 (1.0, 1.4)	−3.43	<0.001	1.2 (1.0, 1.6)	1.3 (1.1, 1.4)	−0.03	0.975
FPG [mmoL/L, M (*Q*_1_, *Q*3)]	5.4 (5.0, 7.0)	5.8 (5.2, 7.5)	−2.32	0.021	5.8 (5.0, 7.3)	5.6 (5.2, 7.5)	−0.07	0.944
HCY [mmoL/L, M (*Q*_1_, *Q*_3_)]	20 (17, 24)	22 (19, 24)	−2.12	0.034	21 (17, 25)	20 (16, 24)	−0.96	0.335
Infarction site (*n*, %)			2.58	0.765			3.51	0.623
Pons	30 (30.0)	60 (28.6)			20 (31.7)	28 (44.4)		
Cerebellum	25 (25.0)	40 (19.0)			18 (28.6)	17 (27.0)		
Thalamus	17 (17.0)	35 (16.7)			12 (19.0)	6 (9.5)		
Medulla oblongata	10 (10.0)	30 (14.3)			3 (4.8)	3 (4.8)		
Occipital lobe	10 (10.0)	24 (11.4)			6 (9.5)	6 (9.5)		
Midbrain	8 (8.0)	21 (10.0)			4 (6.3)	3 (4.8)		
Baseline NIHSS score [score, *M* (*Q*_1_, *Q*_3_)]	3.0 (3.0, 5.0)	4.0 (3.0, 5.0)	−3.37	<0.001	4.0 (3.0, 5.0)	4.0 (3.0, 5.0)	−0.44	0.661
24 h NIHSS score [score, M (*Q*_1_, *Q*_3_)]	3.0 (2.0, 4.0)	5.0 (3.0, 6.0)	−5.37	<0.001	3.0 (3.0, 5.0)	4.0 (3.0, 6.0)	−2.16	0.031
7-day NIHSS score [score, M (*Q*_1_, *Q*_3_)]	3.0 (2.0, 4.0)	4.0 (3.0, 5.0)	−5.47	<0.001	3.0 (2.0, 4.0)	4.0 (2.0, 5.0)	−1.77	0.076
NIHSS score at discharge [score, *M* (*Q*_1_, *Q*_3_)]	2.0 (1.0, 3.0)	3.0 (2.0, 4.0)	−5.53	<0.001	2.0 (1.0, 3.0)	3.0 (2.0, 4.0)	−2.23	0.026

DWI, diffusion-weighted imaging; PCIS, posterior circulation ischemic stroke; PSM, propensity score matching; TC, total cholesterol; TG, triglycerides; LDL-C, low-density lipoprotein cholesterol; HDL-C, high-density lipoprotein cholesterol; FPG, fasting plasma glucose; HCY, homocysteine; NIHSS, National Institutes of Health Stroke Scale.

### 3.2 Clinical outcomes of the patient groups with DWI-negative PCIS without PSM

The independent variable in the multivariate logistic regression analysis was the occurrence of intravenous thrombolysis (the control and thrombolysis groups were assigned 0 and 1, respectively). The primary and secondary outcome measures were the dependent variables. The analysis showed that 43 patients (13.9%) experienced END, and its proportion in the thrombolysis group was lower than that in the control group, with the difference being significant (*p* = 0.002). The proportion of patients with good functional prognosis (mRS ≤ 1) in the thrombolysis group was significantly higher than that in the control group (*p* < 0.001). A higher proportion of patients with mRS ≤ 2 was included in the thrombolysis group than in the control group. Patients with thrombolysis showed a reduced incidence of mRS ≥ 4; however, no significant intergroup differences were observed (*p* > 0.05). No deaths occurred in the thrombolysis group during the 90-day follow-up. By contrast, three patients (1.4%) died in the control group. Two died from stroke-induced pulmonary infection, and one died from upper gastrointestinal bleeding. Two patients (2.0%) in the thrombolysis group developed sICH, whereas no cases of sICH were observed in the control group (Table 2).

**Table 2:** Clinical outcomes of two groups of patients with DWI-negative PCIS without PSM.

Project	Thrombolytic group (*n* = 100)	Control group (*n* = 210)	Binary logistic regression analysis	
			OR value (95% CI)	*p-*value
Primary outcomes [*n* (%)]				
mRS ≤ 1 point after 90 days of onset	80 (80.0)	120 (57.1)	0.333 (0.190–0.584)	<0.001
END	4 (4.0)	39 (18.6)	5.474 (1.898–15.782)	0.002
Secondary outcomes [*n* (%)]				
mRS ≤ 2 points after 90 days of onset	89 (89.0)	160 (76.2)	0.560 (0.208–1.511)	0.253
mRS ≥ 4 points within 90 days of onset	6 (6.0)	34 (16.2)	1.801 (0.506–6.414)	0.364
sICH	2 (2.0)	0	–	–
Mortality	0	3 (1.4)	–	–

DWI, diffusion-weighted imaging; PCIS, posterior circulation ischemic stroke; PSM, propensity score matching; MRS, modified Rankin scale; END, early neurological deterioration; sICH, symptomatic intracranial hemorrhage; –, no data.

### 3.3 Clinical outcomes of the patient groups with DWI-negative PCIS after PSM

After PSM, the incidence of END in the TNK group was lower than that in the control group [1.6% (1/63) vs. 19.0% (12/63)]. Binary logistic regression analysis showed a significant trend of association between TNK treatment and lower END risk [odds ratio (OR) = 0.069, 95% confidence interval (CI): 0.009–0.545, *p* = 0.011]. The proportion of mRS ≤ 1 in the thrombolysis group was significantly higher than that in the control group (*p* = 0.028). The mRS ≥ 4 scores in the thrombolysis group were lower than those in the control group and showed a higher proportion of favorable outcomes (mRS ≤ 2). However, these intergroup variations were not significant (*p* > 0.05). During the 90-day follow-up window, one case (1.6%) of sICH occurred in the thrombolysis group, while no sICH was noted in the control group. Two deaths were recorded during the same period, equivalent to a mortality rate of 3.2%, both due to aspiration pneumonia; however, a direct causal relationship with the index stroke could not be definitively established. Owing to zero or near-zero events in at least one group, the adjusted ORs for sICH and mortality could not be reliably estimated (Table 3).

**Table 3:** Clinical outcomes of two groups of patients with DWI-negative PCIS after PSM.

Project	Thrombolytic group (*n* = 63)	Control group (*n* = 63)	Binary logistic regression analysis	
			OR value (95% CI)	*p-*value
Primary outcomes [*n* (%)]				
mRS ≤ 1 point after 90 days of onset	54 (85.7)	37 (58.7)	0.279 (0.090–0.870)	0.028
END	1 (1.6)	12 (19.0)	0.069 (0.009–0.545)	0.011
Secondary outcomes [*n* (%)]				
mRS ≤ 2 points after 90 days of onset	59 (93.7)	49 (77.8)	2.032 (0.292–14.165)	0.474
mRS ≥ 4 points within 90 days of onset	2 (3.2)	12 (19.0)	2.589 (0.304–22.064)	0.384
sICH	1 (1.6)	0	–	–
Mortality	0	2 (3.2)	–	–

DWI, diffusion-weighted imaging; PCIS, posterior circulation ischemic stroke; PSM, propensity score matching; MRS, modified Rankin scale; END, early neurological deterioration; sICH, symptomatic intracranial hemorrhage; – no data.

## 4 Discussion

PCIS is a cerebral infarction that occurs in the vertebral basilar artery system, and it is responsible for 20% of all ischemic strokes (10). A quarter of the cerebral blood flow is supplied to the brainstem, cerebellum, thalamus, occipital lobe, and temporal lobe via the vertebral basilar artery system. Occlusion of the posterior circulation often accumulates in areas such as the brainstem and posterior inferior cerebellar artery, seriously affecting patient safety (11). The clinical manifestations of PCIS are diverse, with common symptoms including dizziness, diplopia, articulation disorders, and ataxia (12). There is an urgent need for a rapid identification method to avoid delaying early diagnosis and treatment because some patients with acute vestibular syndrome often present with dizziness, which can easily be confused with PCIS. Bedside examination with HINTS distinguishes peripheral vestibular dysfunction from PCIS and even outclasses MRI-DWI in the early stages of PCIS. Head pulse, gaze-induced nystagmus, and eye deviation tests are the primary examination methods (13, 14). Furthermore, bedside examination with HINTS has a higher sensitivity and specificity that MRI in distinguishing between PCIS and peripheral vestibular dysfunction, with a sensitivity of up to 100% and specificity of up to 96% (15). Therefore, bedside examinations are considered in our inclusion criteria. MRI has become a commonly used tool for diagnosing AIS, and DWI can improve our understanding of PCIS with the rapid development of neuroimaging. However, 12%–50% of patients with AIS still have false-negative DWI results (16) for the following reasons: (1) hypoperfusion may cause clinical symptoms of neurological deficits in patients with mild stroke in cases of mild reduction in cerebral blood flow, but it is not sufficient to produce significant changes on DWI (17); (2) the narrow time window between stroke onset and neuroimaging assessment may compromise the DWI efficacy in identifying posterior circulation abnormalities, especially in the hyperacute phase; and (3) brainstem lesions are often small and may produce artifacts that are overlooked (18).

For patients diagnosed with AIS, intravenous thrombolysis should be conducted following the guidelines for the diagnosis and treatment of AIS within the thrombolysis time window to improve ischemia–reperfusion, and short-term dual antiplatelet therapy, intensive statin therapy, and drugs to control vascular risk factors should be actively administered. However, the treatment methods for patients with DWI-negative PCIS are currently unclear. It is theoretically believed that intravenous thrombolysis has a therapeutic effect on patients with DWI-negative PCIS based on the pathogenesis of PCIS (19).

TNK has a longer half-life and higher fibrin specificity as a variant of recombinant tissue plasminogen activator (rt-PA); it is more efficient and easier to administer than rt-PA, which significantly improves the thrombolysis rate in patients before and after hospital transfer (20, 21). The TASTE study showed that TNK was not inferior to rt-PA in patients with AIS within 4.5 h of onset (22). EXTEND-IA TNK study included 204 patients with large vessel occlusion who met the criteria for intravenous thrombolysis and endovascular treatment within 4.5 h of onset. One group received TNK treatment, while the other group received alteplase treatment. The main outcome was defined as angiography evaluation of the relevant ischemic area, reperfusion rate >50%, or no recoverable thrombus. The functional outcomes of the TNK group were found to be better than those of the alteplase group 90 days after treatment, and the TNK intravenous thrombolysis reperfusion rate was better than that of the alteplase group before intravascular treatment (23). In this study, we used TNK at standard doses for intravenous thrombolysis, and the results showed that the proportion of mRS ≤ 1 in the thrombolysis group increased (*p* < 0.05) compared to the scores of patients who received dual antiplatelet aggregation therapy. The incidence of END in patients with DWI-negative PCIS also reduced without increase in mortality. Seyhan et al. (24) included 43 participants with AIS and divided them into DWI-negative (11 patients) and DWI-positive (32 patients) groups based on their DWI results. The patients in both the groups were treated with rt-PA intravenous thrombolysis. The results showed that the proportion of clinical outcomes (mRS ≤ 2) in the DWI-negative group was higher than that in the DWI-positive group after 90 days. Furthermore, patients in the DWI positive group had an anterior CIS. In this study, we investigated the TNK efficacy in treating DWI-negative PCIS and discovered that TNK could attenuate the risk of END and enhance long-term patient prognosis. Förster et al. (25) included 30 patients with posterior circulation stroke (PCS) and 198 patients with anterior circulation stroke (ACS) who received rt-PA intravenous thrombolysis treatment. The therapeutic outcomes of thrombolysis in patients with PCS were not inferior to those in patients with ACS. A meta-analysis included 5,146 patients and divided them into a PCS group of 753 patients and an ACS group of 4,393 patients, both receiving rt-PA intravenous thrombolysis treatment. The results showed that the proportion of mRS 0–2 scores in the PCS group was higher than that in the ACS group, and the sICH and mortality rates were lower than those in the ACS group (26). This finding confirms the efficacy and safety of thrombolysis in patients with PCS. After balancing the general situation with PSM, 63 pairs of patients with DWI-negative PCIS were successfully created. The proportion of mRS scores of 0–2 in the matched thrombolysis group was higher than that in the control group after 90 days of follow-up, indicating that intravenous thrombolysis treatment is beneficial for good outcomes. In terms of safety, two (3.2%) patients in the control group died after PSM matching, due to secondary pulmonary infections caused by stroke; one (1.6%) patient in the thrombolysis group developed sICH. No significant difference was observed for safety between the patient groups after matching.

In conclusion, in this study, we discovered that early TNK intravenous thrombolysis treatment for patients with DWI-negative PCIS can significantly reduce END incidence, improve clinical prognosis, and improve safety. This study provides new notions and methods for treating such patients in clinical practice. This study has some limitations. First, it was retrospective, and patients who could not undergo DWI examination for various reasons were excluded. This may have resulted in possible selection bias. Second, the reduction in sample size and potential residual confounding after PSM limit statistical analysis. We will conduct larger and more balanced cohort studies to confirm these preliminary findings. Third, a limitation related to END analysis is that the post-PSM sample size (63 pairs) and small number of END events (1 in TNK group) limited statistical power. Finally, we did not conduct ordinal shift analysis for 90-day mRS, which may have underutilized the ordinal information of functional recovery. In future multicenter studies, we will expand the sample size to ≥300 thrombolysis cases, adopt ordinal analysis to evaluate the overall shift of mRS scores, and further validate the functional improvement effect of thrombolysis across different disability grades.

## Data Availability

The raw data supporting the conclusions of this article will be made available by the authors, without undue reservation.

## Glossary

PCIS: posterior circulation ischemic stroke
DWI: diffusion-weighted imaging
MRI: magnetic resonance imaging
AIS: acute ischemic stroke
NIHSS: National Institutes of Health Stroke Scale
END: early neurological deterioration
TNK: tenecteplase
HINTS: head impulse nystagmus test of skew
CT: computed tomography
HDL-C: high-density lipoprotein cholesterol
LDL: C low-density lipoprotein cholesterol
HCY: homocysteine
FPG: fasting plasma glucose
T1W1: T1-weighted
T2W2: T2-weighted
FLAIR: fluid-attenuated inversion recovery
TR: repetition time
TE: echo time
mRS: modified Rankin Scale
sICH: symptomatic intracranial hemorrhage
PSM: propensity score matching
OR: odds ratio
CI: confidence interval
rt-PA: recombinant tissue plasminogen activator
ACS: anterior circulation stroke
PCS: posterior circulation stroke
